# Supplementary material for: Content-rich biological network constructed by mining PubMed abstracts
Source: BMC Bioinformatics. 2004 Oct 8;5:147. doi: 10.1186/1471-2105-5-147 (PMC528731; doi:10.1186/1471-2105-5-147)
Supplement: Additional File 2 — The original results of the above study (non-essential files are deleted to keep the file size under the limit set by BMC bioinformatics). [file 1471-2105-5-147-S2.bz2 › chilibotAdditionalFile2/dip10/123ID8668348E467/html/MAP2K2_BRAF.html]

 


 **MAP2K2** and **BRAF** 
  
Found 7 abstracts in PubMed, retrieved 7.  
 

 What does Google say? 
 PDF only 
| .edu only 

---

**Interactive relationship** (e.g. stimulation, inhibition, etc)

**Neutral relationship**- RESULTS In the yeast two hybrid interaction assay, mKsr 1 did not bind to either Ras, B Raf  [ **BRAF** ]  or Raf 1, but interacted strongly with both MEK 1 and MEK 2  [ **MAP2K2** ] , activators of MAP kinase.  Ref: 9427625 Curr Biol, 1998

**Non-interactive relationship** (e.g. studied together, co-existance, homology, etc.)

- The first MEK activator has an apparent M r of 4 00 5 00, was immunologically distinct from A Raf, B Raf  [ **BRAF** ] , c Raf 1, c MEKK, c Mos, MEK1, and MEK2  [ **MAP2K2** ] , and was rapidly activated by serum, platelet derived growth factor PDGF, insulin, thrombin, and phorbol ester.  Ref: 7706312 J Biol Chem, 1995
- The prototype mitogen activated protein kinase module is a three kinase cascade consisting of the MAP kinase, extracellular signal regulated protein kinase 1 or ERK2, the MAP ERK kinase MEK1 or MEK2  [ **MAP2K2** ] , and the MEK kinase, Raf 1 or B Raf  [ **BRAF** ] .  Ref: 7499418 J Biol Chem, 1995
- We show that, consequently, B Raf  [ **BRAF** ]  interacts with MEK 1 and MEK 2  [ **MAP2K2** ]  with a better affinity than does c Raf 1, thus strengthening the notion that B Raf  [ **BRAF** ]  is a stronger MEK activator than c Raf l.  Ref: 8668348 Oncogene, 1996
- Receptor induced activation of other Ser Thr kinases, such as c Raf, B Raf  [ **BRAF** ] , Mek1, Mek2  [ **MAP2K2** ] , Mekk, was not affected by wortmannin.  Ref: 8654435 Eur J Biochem, 1996
